# Adverse Reactions to CT Contrast Agents: A 10-Year Study of Clinical and Environmental Risk Factors

**DOI:** 10.3390/diagnostics15222820

**Published:** 2025-11-07

**Authors:** Min-gyu Kim, Hojin Kim, Kwangmin Lee, Wonseok Yang, Eun-ju Kang, Yongsu Yoon

**Affiliations:** 1 Department of Radiology, Dong-A University Hospital, Busan 49201, Republic of Korea; kimmin612@naver.com (M.-g.K.); hojin801812@gmail.com (H.K.); ywsuck@naver.com (W.Y.); medcarrot@dau.ac.kr (E.-j.K.); 2 Department of Multidisciplinary Radiological Sciences, The Graduate School of Dongseo University, Busan 47011, Republic of Korea; 3 Department of Cardiology, Dong-A University Hospital, Busan 49201, Republic of Korea; tnt849@hanmail.net

**Keywords:** computed tomography, contrast agent, adverse drug reaction, environmental factors, premedication, risk factor

## Abstract

**Background**: Iodinated contrast agents are widely used in computed tomography (CT) imaging; however, they can cause adverse drug reactions (ADRs) ranging from mild hypersensitivity to severe anaphylaxis. While several clinical risk factors have been identified, large–scale studies incorporating environmental variables remain limited. This study aimed to assess the prevalence and predictors of contrast agent-related ADRs over a 10-year period. **Methods**: We retrospectively analyzed 221,962 adult outpatients who underwent contrast-enhanced CT between January 2014 and December 2023 at a single tertiary center: Patient characteristics, clinical conditions (e.g., hypertension, allergy history), contrast agent types, premedication status, seasonal trends, temperature, and humidity were examined. ADRs were categorized as mild, moderate, or severe based on American College of Radiology (ACR) guidelines. Logistic regression was used to identify independent predictors. **Results**: The overall prevalence of ADRs was 0.64% (1423 cases). ADRs were more frequent in females, younger patients, and those receiving premedication. Seasonal and environmental patterns were evident: higher ADR rates occurred in summer and autumn, with positive correlations to ambient temperature and humidity. Among contrast agents, Ioversol (1.4%) and Iomeprol (1.2%) showed the highest ADR rates. The prevalence of mild ADRs increased in the post–COVID-19 period, while that of moderate reactions declined. **Conclusions**: This real–world study identified multiple clinical and environmental factors associated with ADRs to iodinated contrast agents in CT imaging. The findings suggest the importance of individualized risk assessment and the consideration of environmental factors when planning contrast administration.

## 1. Introduction

Computed tomography (CT) is a widely used diagnostic modality that enables cross-sectional and three-dimensional imaging through the differential absorption of X-rays by body tissues [1]. To improve diagnostic accuracy, iodinated contrast agents are commonly administered during CT imaging. These agents enhance visualization by differentiating pathological from normal tissues based on their distribution patterns [2].

Iodinated contrast agents are classified into ionic monomers, nonionic monomers, and nonionic dimers, with nonionic formulations being the standard for intravenous use [3]. Although generally safe, contrast agents can induce adverse drug reactions (ADRs), ranging from mild hypersensitivity (e.g., nausea, pruritus) to severe life-threatening events such as anaphylaxis and cardiac arrest. Acute reactions occur within one hour, while delayed reactions may manifest up to seven days post-administration [4,5]. In addition to hypersensitivity, contrast-induced nephropathy remains a concern, particularly in patients with impaired renal function or diabetes mellitus [6].

To minimize these risks, pre-imaging assessments including serum creatinine levels and discontinuation of nephrotoxic medications (e.g., metformin) are recommended. Premedication with antihistamines or corticosteroids is often considered in patients with a history of prior reactions. Despite these measures, predicting ADRs remains challenging due to multifactorial influences.

Recent attention has turned to environmental variables such as ambient temperature and humidity, which may modulate physiological responses and immune reactivity [7,8]. Furthermore, changes in clinical practice and immune status during the COVID-19 pandemic may also affect ADR prevalence [9,10]. However, real-world, large-scale studies that simultaneously evaluate clinical and environmental predictors of contrast-related ADRs are limited.

This study aimed to evaluate the prevalence and characteristics of ADRs following iodinated contrast agent administration over a 10-year period and to identify associated clinical and environmental risk factors. These findings are expected to support safer, personalized approaches to contrast use in CT imaging.

## 2. Materials and Methods

### 2.1. Study Design and Population

This study was approved by the Institutional Review Board of Dong-A University Hospital (protocol code DAUHIRB-24-068 and date of approval: 17 April 2024). We analyzed the prevalence and associated factors of ADRs in outpatients who received contrast agents during CT examinations over a 10-year period. Only adult patients aged 18 years or older were included, and patients with incomplete data or duplicate records were excluded. Inpatients and emergency cases were excluded because their conditions differ substantially from those of outpatients. These groups often present with higher disease severity, multiple comorbidities, and different contrast administration purposes, as well as a greater likelihood of premedication use. This study included 221,962 outpatients who underwent contrast-enhanced CT examinations using whole-body CT scanners (GE Healthcare, Chicago, IL, USA; Siemens Healthineers, Erlangen, Germany; Toshiba Medical Systems, Otawara, Japan) between 1 January 2014, and 31 December 2023. The examinations included various types such as brain, chest, abdomen, and extremities. All outpatients who required contrast-enhanced CT for clinical purposes were included. Among these patients, 1423 individuals who developed acute reactions within one hour after contrast agent administration were included for analysis. ADRs were identified through clinician reporting, within the institutional adverse-reaction reporting system.

### 2.2. Contrast Agent Administration and Premedication

Nonionic CT contrast agents were injected at a rate of 2–5 mL per second, with a total volume of 100–140 mL, according to the scan protocols for each examination site. The contrast agents used in this study included Iomeprol, Iopamidol (Bracco Imaging, Milan, Italy), Iobitridol (Guerbet, Villepinte, France), Iohexol (GE Healthcare, Cork, Ireland), and Ioversol (Guerbet, Villepinte, France). All contrast agents used in this study are low-osmolarity nonionic iodinated contrast agents commonly used for CT. Prior to CT imaging, all patients were informed about the use of contrast agents and provided their consent. For patients with a history of adverse reactions to CT contrast agents, premedication with antihistamines or pheniramine was administered before the examination. Although a standardized institutional protocol for premedication was not established, it was generally administered even to patients with mild prior reactions, and additional medications were provided as deemed necessary at the discretion of the attending physician.

### 2.3. Classification of Adverse Reactions

This study included only acute reactions occurring within one hour of contrast administration. The severity of adverse reactions to CT contrast agents was classified as mild, moderate, or severe according to the American College of Radiology (ACR) Manual on Contrast Media. Categorization was performed by a single reviewer based on these standardized definitions, and inter-observer validation or double review was not required since the classification followed the ACR manual without subjective interpretation. Immune-mediated reactions were identified when clinical features suggested hypersensitivity, such as urticaria, angioedema, bronchospasm, or anaphylaxis. Physiologic (non-immunologic) reactions, including nausea, vomiting, dizziness, or vasovagal responses, were classified separately as physiologic reactions according to ACR definitions. This study included only acute reactions occurring within one hour of contrast administration.

Mild reactions included allergic responses such as localized urticaria, pruritus, edema, and rhinorrhea, as well as physiologic responses such as nausea, vomiting, and dizziness. Symptoms or signs are perceptible but easily tolerable, and they resolve spontaneously without evidence of progression.

Moderate reactions included allergic responses such as generalized urticarial, pruritus, bronchospasm, and hypoxia, along with physiologic responses such as hypertension and chest pain. Symptoms were sufficiently uncomfortable to interfere with daily activities, require medical treatment, and could potentially worsen if left untreated.

Severe reactions included allergic responses such as respiratory distress, severe hypoxia, and anaphylactic shock, and physiologic responses such as arrhythmia, convulsions, or seizures. Severe reactions require intensive medical intervention and could result in permanent sequelae or pose a threat to life.

### 2.4. Environmental Factors and COVID-19 Period Classification

Daily temperature and humidity data were obtained from the Korea Meteorological Administration. To evaluate the association between COVID-19 and contrast agent-related adverse reactions, the study period was divided into three phases: pre-pandemic, pandemic, and post-pandemic. The pandemic period was defined based on the WHO declaration of COVID-19 as a global pandemic. Specifically, the pre-pandemic period was defined as January 2014 to 10 March 2020; the pandemic period as 11 March 2020 to 5 May 2023; and the post-pandemic period as 6 May 2023 to December 2023 [9,10].

### 2.5. Statistical Analysis

Statistical analysis was performed using SAS version 9.4 (SAS Institute, Cary, NC, USA), and the specific statistical methods employed are described as follows. Categorical variables were presented as frequencies and percentages, and continuous variables were presented as means and standard deviations or as medians and interquartile ranges (IQRs), depending on data distribution. For comparisons between groups according to the presence or absence of adverse reactions, categorical variables were analyzed using the chi-square test or Fisher’s exact test, and continuous variables were analyzed using the independent *t*-test or the Mann-Whitney U-test. Predictors of adverse reactions were identified using logistic regression analysis with the backward elimination method. Statistical significance was set at a two-sided *p* < 0.05. Prevalence of adverse reactions was calculated using binomial proportions with 95% confidence intervals. Confidence intervals were estimated using the Wilson method.

## 3. Results

(1) Baseline Characteristics

A total of 221,962 adult outpatients underwent contrast-enhanced CT between January 2014 and December 2023. The overall prevalence of contrast agent-related ADRs was 0.64% (1423/221,962).

As shown in Table 1, the ADR prevalence was significantly higher in patients aged <40 years (1.3%, 189/14,010) compared with those aged ≥70 years (0.4%, 252/64,020) (*p* < 0.001). Female patients (0.8%, 776/93,433) showed a higher prevalence than males (0.5%, 647/128,529) (*p* < 0.001). Patients with a history of allergies exhibited a higher ADR rate (1.8%, 5/279) compared to those without (0.6%, 1418/221,683) (*p* = 0.016). Patients with hypertension had a lower prevalence of ADRs (0.4%, 224/50,055) than those without (0.7%, 1199/171,907) (*p* < 0.001). There was no significant difference in ADR prevalence based on asthma, diabetes, hyperlipidemia, or cardiovascular/neurologic disease.

Premedicated patients had a higher ADR rate (4.0%, 203/5051) than those without premedication (0.6%, 1220/216,911) (*p* < 0.001).

Among the five types of contrast agents, Ioversol (1.4%, 14/1029) and Iomeprol (1.2%, 69/5531) showed the highest ADR prevalence (*p* < 0.001).

(2) Temporal Trends in ADR prevalence

**Figure 1:**
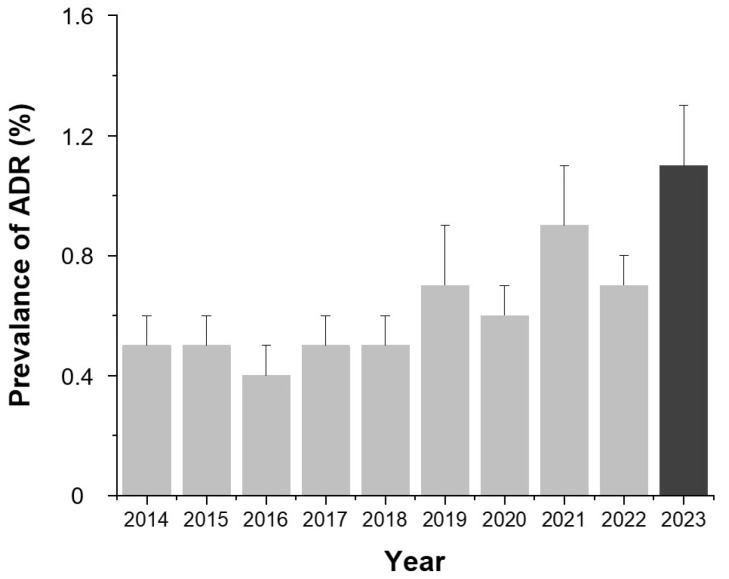
Annual prevalence of contrast agent-related ADRs from 2014 to 2023. Bars indicate the mean prevalence (%) for each year and error bars indicate 95% confidence intervals calculated using the Wilson method. Values are presented as number (percentage). Abbreviation: ADR, adverse drug reaction.

As illustrated in Figure 1, the annual ADR prevalence showed an increasing trend from 2014 to 2023, rising from 0.5% (95% CI, 0.4–0.6%) in 2014 to 1.1% (95% CI, 1.0–1.2%) in 2023, with slight fluctuations in 2016, 2020, and 2022.

**Figure 2:**
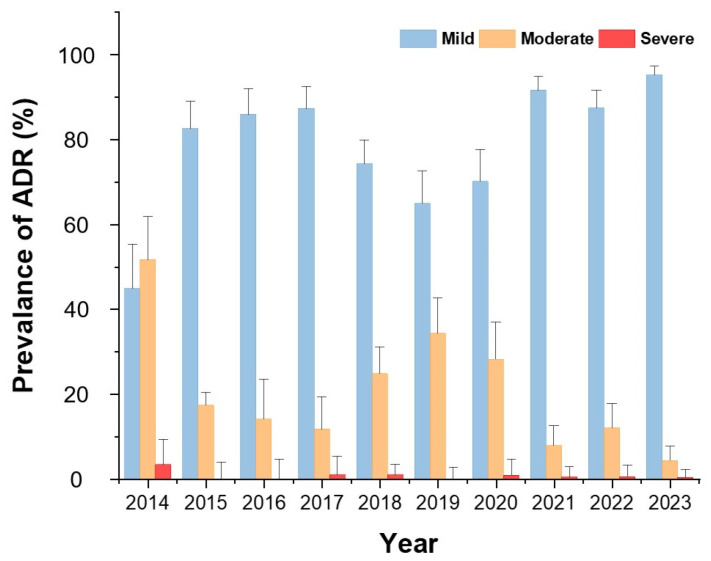
Annual distribution of ADR severity from 2014 to 2023. Bars indicate the proportion (%) of mild, moderate, and severe reactions among all ADR cases.

Figure 2 demonstrates that mild reactions accounted for the majority of ADRs across all years except 2014. The proportion of severe ADRs remained low throughout the study period.

**Figure 3:**
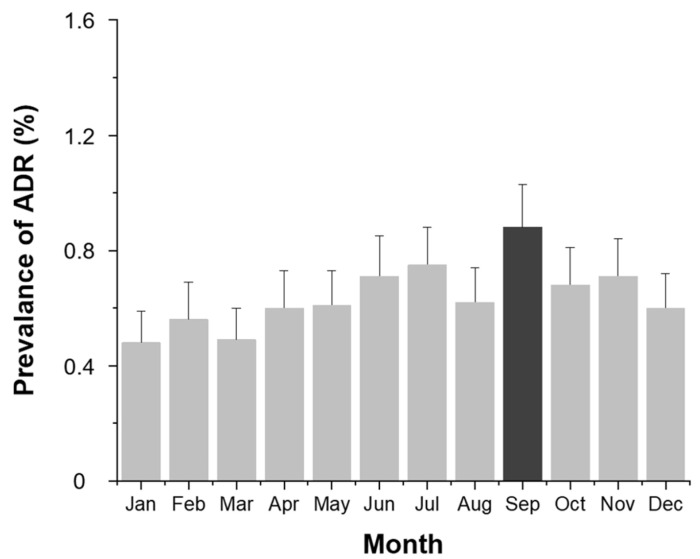
Monthly prevalence (%) of contrast agent-related ADRs. Bars indicate the observed proportion of ADR cases among all examinations for each month.

Monthly analysis (Figure 3) revealed higher ADR rates in June, July, September, and November, corresponding to the summer and autumn seasons (*p* < 0.001).

(3) Environmental Factors: Temperature and Humidity

**Figure 4:**
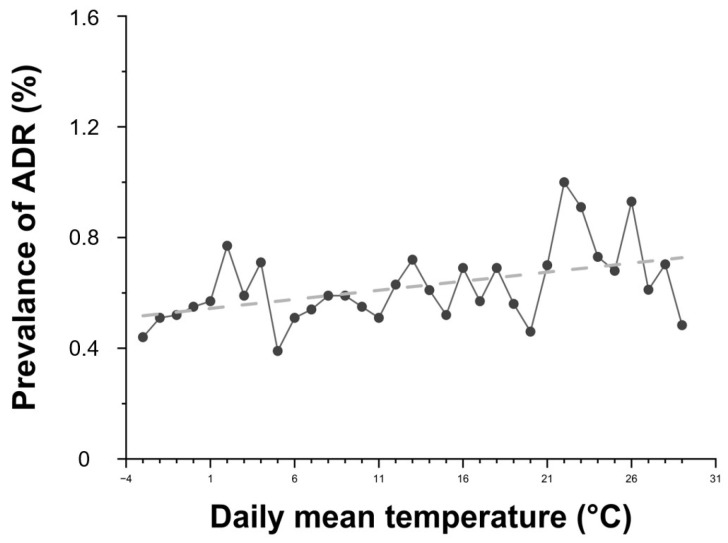
Relationship between daily average temperature and the prevalence of contrast agent-related ADRs. Black dots represent the prevalence of ADRs according to daily mean temperature, and the dashed line indicates the linear regression trend (R^2^ = 0.2134). R^2^ = 0.2134 indicates the proportion of variance in ADR prevalence explained by daily mean temperature (linear fit).

**Figure 5:**
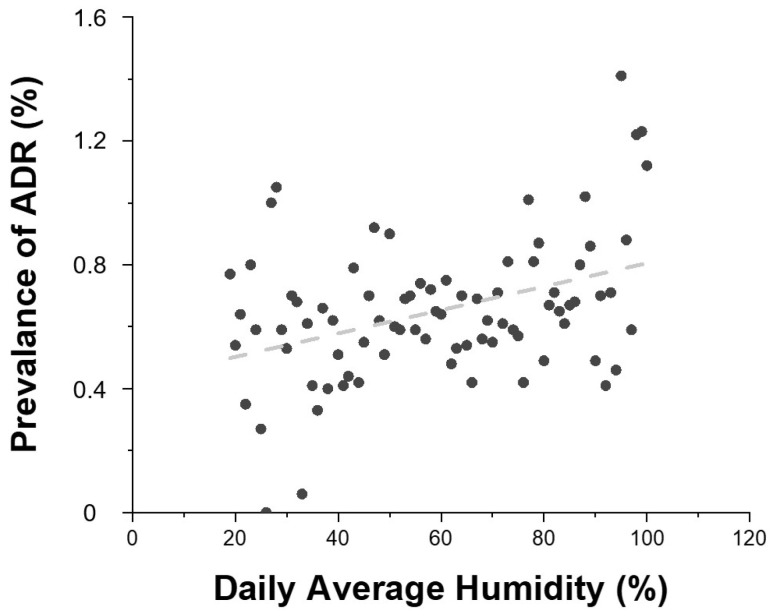
Relationship between daily average humidity and the prevalence of contrast agent-related ADRs. Gray dots represent the prevalence of ADRs according to daily mean humidity, and the dashed line indicates the linear regression of the trend (R^2^ = 0.155). R^2^ = 0.155 indicates the proportion of variance in ADR prevalence explained by daily average humidity (linear fit).

Daily average temperature showed a positive correlation with ADR prevalence (Figure 4). As the temperature increased, so did the prevalence of ADRs. Higher average humidity levels were also associated with increased ADR risk (Figure 5).

(4) Contrast Agent Type

**Figure 6:**
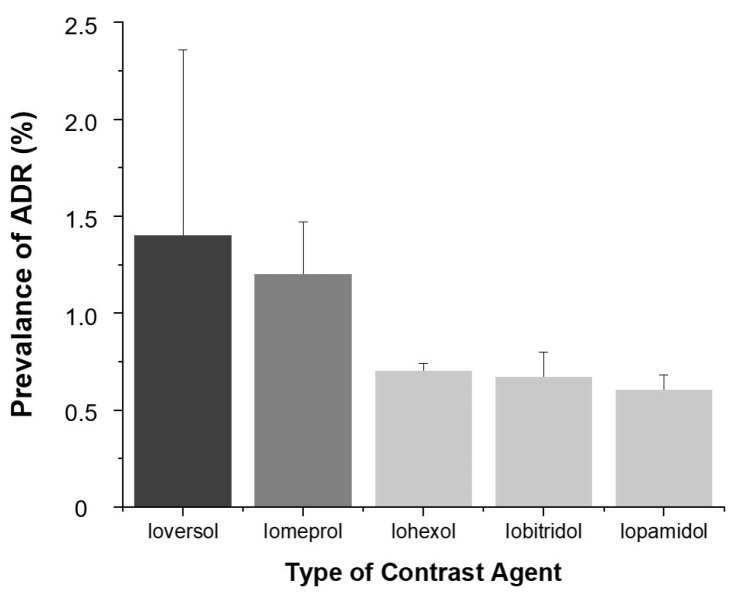
Prevalence of ADRs according to contrast agent. Bars indicate ADR prevalence (%) and error bars represent 95% confidence intervals.

As shown in Figure 6, the prevalence of ADRs varied by contrast agent. Ioversol (1.4%) and Iomeprol (1.2%) demonstrated significantly higher ADR rates compared to Iohexol (0.7%), Iobitridol (0.7%), and Iopamidol (0.6%) (*p* < 0.001).

(5) COVID-19 Pandemic Period Comparison

When comparing the pre-pandemic, pandemic, and post-pandemic periods of COVID-19, mild ADRs were consistently accounted for the highest proportion, with an overall increasing trend observed from the onset of the pandemic compared to the pre-pandemic period. In contrast, the prevalence of moderate ADRs showed a decreasing trend starting from the pandemic period. The prevalence of severe ADRs showed no significant change over time (Figure 2).

(6) Association with Premedication

The ADR rate was significantly higher in patients who received premedication (4.0%) compared to those who did not (0.6%) (Table 1).

(7) Multivariate Logistic Regression Analysis

Female patients had a higher risk of ADRs compared to males (OR = 1.536, *p* < 0.001), and younger age groups, particularly those under 40 years, showed significantly elevated risks compared to the ≥70 age group (OR = 3.170, *p* < 0.001). A history of allergy (OR = 2.770, *p* = 0.025) and diabetes (OR = 1.268, *p* = 0.008) were also associated with increased ADR risk, whereas hypertension was linked to a reduced risk (OR = 0.726, *p* < 0.001). Seasonal variation was evident, with ADR prevalence significantly higher in summer and autumn compared to winter (OR = 1.269 and 1.367, respectively; *p* < 0.01). Pre-medication was significantly associated with ADR occurrence (OR = 7.409, *p* < 0.001). Among contrast agents, Iomeprol (OR = 2.399) and Ioversol (OR = 1.962) were linked to higher ADR risk compared to Iopamidol, while Iobitridol showed no significant difference (Table 2).

The overall model performance was evaluated using the Hosmer–Lemeshow goodness-of-fit test (χ^2^ = 7.41, *p* = 0.492) and the area under the ROC curve (AUC = 0.771).

## 4. Discussion

This 10-year retrospective study analyzed a large cohort of patients undergoing contrast-enhanced CT to investigate the prevalence and predictors of iodinated contrast agent-related ADRs. The overall prevalence of ADRs was 0.64%, which is comparable to the reported prevalence of acute adverse reactions to nonionic low-osmolar contrast agents (0.2–0.7%) by the ACR [11]. The majority of reactions were mild, and the prevalence of severe ADRs remained very low throughout the study period.

Although recent pharmacological advances have improved the safety profiles of contrast agents, the prevalence of ADRs nearly doubled over the 10-year study period, increasing from 0.5% in 2014 to 1.1% in 2023. This apparent upward trend may not necessarily indicate a true increase in reaction risk but could instead reflect several contextual factors. First, institutional monitoring systems and staff awareness have improved, leading to more comprehensive documentation of mild or transient reactions that may have been underreported in earlier years. Second, the patient population has gradually aged, and a greater proportion of individuals with various chronic conditions have undergone contrast-enhanced imaging, which could partially explain the upward trend in ADR prevalence. Third, the total number of examinations performed annually has substantially increased, thereby increasing the likelihood of detecting minor or self-limiting reactions. Collectively, these factors suggest that the observed increase in ADR prevalence is more likely attributable to enhanced detection and reporting rather than a deterioration in the pharmacologic safety of modern contrast agents.

Also, this study was conducted in an outpatient setting. Outpatients are generally in a relatively stable condition when undergoing imaging examinations, and the prevalence of adverse reactions to contrast agents has been reported to be low in this population [12]. This contrasts with inpatients or emergency patients, who often have greater disease severity and comorbidities, and thus the findings may serve as clinical evidence supporting the safety of contrast agent use in the outpatient environment. In addition, patients aged <18 years were excluded due to physiological differences and variations in diagnostic criteria.

Several patient-related factors were independently associated with ADR occurrence. Younger age and female sex emerged as strong predictors, aligning with prior studies that suggest heightened immune responsiveness and hormonal modulation as contributing factors [13,14]. Notably, patients without hypertension had a higher prevalence of ADRs than those with hypertension. This may reflect the vasodilatory effects of antihypertensive agents such as angiotensin-converting enzyme (ACE) inhibitors or calcium channel blockers, as well as immunological differences in the hypertensive population [15].

This study also highlighted significant associations between ADR prevalence and environmental factors. The prevalence of ADRs was higher in summer and autumn, and elevated temperature and humidity showed a positive correlation with ADR risk. However, seasonal variation is unlikely to have substantially confounded these associations, as the number of patients undergoing contrast-enhanced CT was relatively consistent across seasons (Winter: 55,121; Spring: 54,865; Summer: 56,665; Autumn: 55,311). Although the seasonal distribution of contrast agent types could not be confirmed, all examinations were performed within a single institution under standardized imaging protocols, minimizing the potential influence of seasonal differences in patient volume or contrast agent selection. These findings are similar to previous studies [12] and may be interpreted as the result of a combined effect of environmental factors and physiological changes. Also, the findings support the hypothesis that thermal stress, dehydration, vasodilation, and immune system modulation may influence contrast sensitivity [8,16,17].

During the summer, dehydration caused by fluid loss due to high temperature and humidity, along with peripheral vasodilation and decreased blood pressure induced by heat, may contribute to reducing renal blood flow and glomerular filtration rate (GFR), potentially leading to delayed excretion of contrast agents [18,19]. Increased outdoor activities and intense ultraviolet (UV) exposure during the summer could be potential contributing factors to hypersensitivity reactions to contrast agents. Although this mechanism has not been empirically validated, UV radiation-induced skin and immune activation may represent one of several possible explanations for the observed seasonal variation. These interpretations should therefore be considered speculative. In autumn, large diurnal temperature variations and dry weather may lead to reduced body fluid levels, resulting in electrolyte imbalance and impaired renal function, which could contribute to the occurrence of contrast agent-related adverse reactions [8]. Importantly, our use of real-world environmental data from a large clinical population adds robust evidence to this underexplored area.

We also examined ADR patterns before and after the COVID-19 pandemic. A relative increase in mild ADRs was observed in the post-pandemic period, while moderate and severe reactions remained stable. This may be attributable to altered immune profiles, vaccination effects, changes in health-seeking behaviors, or increased awareness/reporting during the pandemic era [20,21]. Although speculative, these trends warrant continued surveillance in post-pandemic healthcare systems.

Among contrast agents, Ioversol and Iomeprol were associated with higher ADR rates compared to Iohexol, Iopamidol, and Iobitridol. These findings are consistent with previous findings reporting a relatively higher prevalence of adverse reactions associated with Iomeprol [22,23,24]. These differences may be attributable to the physicochemical properties of each contrast agent, such as chemical structure, viscosity, and osmolality. Although our study did not directly measure physicochemical properties, such factors likely underlie inter-agent variability and deserve further investigation. Historically, the introduction of low-osmolar nonionic contrast agents has represented a major milestone in improving the safety of radiologic examinations. Early prospective and multicenter studies reported that high-osmolar ionic contrast agents showed a markedly higher rate of adverse reactions, with an overall prevalence of approximately 12.7% [25,26]. In contrast, the adverse reaction rate of low-osmolar nonionic contrast agents was approximately 3.1% in both studies. In the present study, the overall prevalence of ADRs was 0.64%, which is consistent with the ADR prevalence for nonionic low-osmolar contrast agents (0.2–0.7%) reported by the ACR [11] and significantly lower than the rates described for ionic agents in earlier decades. These findings clearly illustrate the progressive evolution of contrast agent safety over time, reflecting the transition from high-osmolar ionic to modern low-osmolar nonionic formulations and the resultant decline in both frequency and severity of adverse reactions.

Premedication was associated with a substantially higher prevalence of ADRs, which likely reflects indication bias, as it was predominantly administered to patients with a known history of contrast sensitivity. The higher ADR prevalence observed among premedicated patients may indeed reflect indication bias, as premedication is typically administered to individuals with a prior history of contrast reactions or higher clinical risk profiles. Therefore, the observed association does not necessarily indicate a causal relationship between premedication and ADR occurrence, but rather reflects the characteristics of the population receiving prophylactic treatment. Nonetheless, this finding underscores the importance of careful patient selection and individualized premedication strategies in line with ACR recommendations [11].

From a clinical perspective, our results suggest that risk assessment for contrast administration should consider not only traditional patient factors but also environmental conditions such as season, temperature, and humidity. In high-risk scenarios–such as summer months or heatwaves—enhanced hydration, monitoring, or premedication protocols may be warranted.

This study has several limitations. First, the retrospective design introduces inherent limitations related to documentation accuracy and reporting bias. Second, the post-pandemic observation period was relatively short, limiting our ability to assess long-term trends. Third, we did not evaluate the impact of repeated contrast exposure or account for individual contrast dose, which may affect reaction likelihood. In addition, certain potential confounders-such as patients’ hydration status, concurrent medications, and detailed comorbid conditions-could not be assessed or controlled because these data were not available for outpatients undergoing CT examinations. Moreover, explicit seasonal adjustment was not performed in the regression model, although day-of-week and meteorological variables such as temperature and humidity may have indirectly captured part of the seasonal variation. Lastly, the study population was derived from a single tertiary center, which may limit generalizability.

Despite the limitations, our study represents one of the largest single-center analyses of contrast agent-related ADRs to date, integrating both clinical and environmental variables in a real-world setting. These findings can inform the development of risk stratification models and contrast safety protocols tailored to both patient characteristics and external conditions.

Future research should focus on the development and validation of predictive models incorporating demographic, clinical and meteorological data to enhance individualized risk assessment. Prospective studies are also needed to evaluate whether adjusting contrast administration protocols according to environmental conditions can reduce ADR prevalence and improve patient safety.

## 5. Conclusions

Age and sex (general characteristics), temperature (environmental factor), and hypertension status (clinical condition) were significantly associated with the prevalence of contrast agent-related adverse reactions. In addition, a strong association was observed between adverse reaction rates and both the type of contrast agent and the administration of premedication.

Notably, fluctuations in temperature and humidity may influence immune responses and fluid balance in the human body. These findings suggest that contrast agent-related adverse reactions may be affected not only by individual patient characteristics but also by external environmental conditions.

## Figures and Tables

**Table 1:** Baseline characteristics.

Total (n)		221,962		
Variable	Categories	ADR		*p*-Value
		No (n = 220,539)	Yes (n = 1423)	
Sex	Male	127,882 (99.5)	647 (0.5)	<0.001
	Female	92,657 (99.2)	776 (0.8)	
Age	<40	13,821 (98.7)	189 (1.3)	<0.001
	40–49	21,321 (99.0)	213 (1.0)	
	50–59	49,923 (99.2)	380 (0.8)	
	60–69	71,706 (99.5)	389 (0.5)	
	≥70	63,768 (99.6)	252 (0.4)	
Asthma	No	219,105 (99.4)	1415 (0.6)	0.686
	Yes	1434 (99.4)	8 (0.6)	
Allergy	No	220,265 (99.4)	1418 (0.6)	0.016
	Yes	274 (98.2)	5 (1.8)	
Hypertension	No	170,708 (99.3)	1199 (0.7)	<0.001
	Yes	49,831 (99.6)	224 (0.4)	
Diabetes	No	191,526 (99.4)	1252 (0.6)	0.205
	Yes	29,013 (99.4)	171 (0.6)	
Hyperlipidemia	No	214,785 (99.4)	1394 (0.6)	0.178
	Yes	5754 (99.5)	29 (0.5)	
Cardiovascular disease	No	207,222 (99.4)	1353 (0.6)	0.070
	Yes	13,317 (99.5)	70 (0.5)	
Neurological disease	No	206,709 (99.4)	1349 (0.6)	0.067
	Yes	13,830 (99.5)	74 (0.5)	
Season	Winter	54,818 (99.5)	303 (0.5)	<0.001
	Spring	54,554 (99.4)	311 (0.6)	
	Summer	56,271 (99.3)	394 (0.7)	
	Autumn	54,896 (99.2)	415 (0.8)	
Pre-Medication	No	215,691 (99.4)	1220 (0.6)	<0.001
	Yes	4848 (96.0)	203 (4.0)	
Type of contrast agent	Iopamidol	130,892 (99.4)	743 (0.6)	<0.001
	Iohexol	51,796 (99.3)	386 (0.7)	
	Iomeprol	5462 (98.8)	69 (1.2)	
	Ioversol	1015 (98.6)	14 (1.4)	
	Iobitridol	31,374 (99.3)	211 (0.7)	

Values are presented as number (percentage). *p* < 0.05 was considered statistically significant. Abbreviation: ADR, adverse drug reaction.

**Table 2:** Multivariate Logistic Regression Analysis of Factors Associated with Contrast Agent-Related Adverse Reactions.

Variable	Categories	Odds Ratio	95% CI		*p*
			Lower	Upper	
Sex	Male	Reference			
	Female	1.536	1.380	1.709	<0.001
Age	<40	3.170	2.603	3.861	<0.001
	40–49	2.238	1.851	2.705	<0.001
	50–59	1.751	1.487	2.062	<0.001
	60–69	1.326	1.130	1.556	<0.001
	≥70	Reference			0.001
Allergy	No	Reference			
	Yes	2.770	1.134	6.766	0.025
Hypertension	No	Reference			
	Yes	0.726	0.619	0.852	<0.001
Diabetes	No	Reference			
	Yes	1.268	1.064	1.511	0.008
Season	Winter	Reference			<0.001
	Spring	1.034	0.882	1.212	0.684
	Summer	1.269	1.091	1.475	0.002
	Autumn	1.367	1.178	1.587	<0.001
Pre-Medication	No	Reference			
	Yes	7.409	6.347	8.649	<0.001
Type of contrast agent	Iopamidol	Reference			<0.001
	Iohexol	1.280	1.130	1.449	<0.001
	Iomeprol	2.399	1.869	3.079	<0.001
	Ioversol	1.962	1.148	3.354	0.014
	Iobitridol	0.972	0.832	1.136	0.723

## Data Availability

The data presented in this study are not available because they are owned and managed by Dong-A University Hospital and cannot be shared under the hospital’s privacy and confidentiality regulations.

## Abbreviations

The following abbreviations are used in this manuscript:

CT	Computed Tomography
ADR	Adverse Drug Reaction
